# Evaluation of mental health using MHC-SF in patients with paget’s bone disease

**DOI:** 10.1192/j.eurpsy.2024.1234

**Published:** 2024-08-27

**Authors:** A. Feki, I. Sellami, C. Abid, A. Abbes, Z. Gassara, S. Ben jemaa, M. Ezzeddine, M. H. Kallel, H. Fourati, R. Akrout, S. Baklouti

**Affiliations:** ^1^Rheumatology; ^2^occupational medicine, Hedi chaker hospital, Sfax, Tunisia

## Abstract

**Introduction:**

Paget’s disease is a chronic bone disorder, that is characterized by increased and disorganized bone remodelling, which can lead to bone pain, bone complications such as deformities and fractures, neurological and cardiovascular complications. This physical impact can alter patients ‘mental health and lead to anxiety or depression.

**Objectives:**

This study aimed to assess the mental health in patients diagnosed with paget’s bone disease

**Methods:**

Paget’s disease patients were assessed by The Mental Health Continuum Short Form (MHC-SF) score. It consists of 14 items that were selected to represent each fact of well-being: 3 emotional well-being items (reflects hedonic well-being), 6 psychological well-being items, and 5 social well-being items (when combined, reflects eudemonic well-being). Items scores are summed, yielding a total score ranging from 0 to 70. Higher scores indicate greater levels of positive well-being.

**Results:**

Thirty patients were included. 60% were men and 40% were women. The average age was 65 years. Socio-economic level was low in 3.3%, average in 86.7%, good in 10% of cases. 93.3% were married and 6.7% were single. For the medical history, 80% had a previous history and 20% did not. Clinically, 83.3% had pain and 16.7% had no pain. Concerning the disease location, 4 had involvement of the skull, 15 of the spine, 13 of the sacrum, 13 of the femur, 1 of the tibia, 1 of the calcaneus and 3 of the humerus. As for complications, 36.7% had no complications, 56.7% had osteoarticular complications, 3.3% had neurological complications and 3.3% had cardio-vascular complications. Concerning treatment, 90% received bisphosphonate and 10% did not.

For the mental health questionnaire, the mean score was 36.4.

53.3% of patients had poor mental health, 43.3% were moderately healthy and 3.3% were thriving.

No significant associations were noted between level of mental health and age, pain level, complications, location of the disease, alkaline phosphatase and treatment p>0.05.

**Conclusions:**

The impact of paget’s disease is not only physical but also psychological. The MHC-SF is useful to detect the mental illness.

**Disclosure of Interest:**

None Declared

